# Distinct progression patterns of brain disease in infantile and juvenile gangliosidoses: Volumetric quantitative MRI study

**DOI:** 10.1016/j.ymgme.2017.12.432

**Published:** 2017-12-20

**Authors:** Igor Nestrasil, Alia Ahmed, Josephine M. Utz, Kyle Rudser, Chester B. Whitley, Jeanine R. Jarnes-Utz

**Affiliations:** a Department of Pediatrics, University of Minnesota, Minneapolis, MN, USA; b Division of Biostatistics, University of Minnesota, Minneapolis, MN, USA; c Advanced Therapies Department, University of Minnesota, Fairview, Minneapolis, MN, USA; d Gene Therapy Center, Department of Pediatrics, University of Minnesota, Minneapolis, MN, USA; e Experimental and Clinical Pharmacology, University of Minnesota, Minneapolis, MN, USA

**Keywords:** β-galactosidase, Gangliosidosis, GM1-gangliosidosis, GM2-gangliosidosis, Tay-Sachs disease, Sandhoff disease

## Abstract

**Background:**

GM1-gangliosidosis and GM2-gangliosidosis (Tay-Sachs disease and Sandhoff disease) are unrelenting heritable neurodegenerative conditions of lysosomal ganglioside accumulation. Although progressive brain atrophy is characteristic, longitudinal quantification of specific brain structures has not been systematically studied.

**Objectives:**

The goal of this longitudinal study has been to quantify and track brain MRI volume changes, including specific structure volume changes, at different times in disease progression of childhood gangliosidoses, and to explore quantitative brain MRI volumetry (qMRI) as a non-invasive marker of disease progression for future treatment trials.

**Methods:**

Brain qMRI studies were performed in 14 patients with gangliosidoses (9 infantile, 5 juvenile) yearly. Cerebellar cortex and white matter, caudate, putamen, corpus callosum, ventricles, total brain, and intracranial volumes were measured, as well as total brain volume. Age-matched controls were available for the patients with the juvenile phenotype.

**Results:**

The infantile phenotype of all gangliosidoses showed a consistent pattern of macrocephaly and rapidly increasing intracranial MRI volume with both (a) brain tissue volume (cerebral cortex and other smaller structures) and (b) ventricular volume (P < 0.01 for all). In contrast to apparent enlargement of the total brain volume, and chiefly the enlarged cerebral cortex, a subset of smaller brain substructures generally decreased in size: the corpus callosum, caudate and putamen became smaller with time. The volume of cerebellar cortex also decreased in patients with infantile GM1-gangliosidosis and juvenile GM1- and GM2-gangliosidosis; however, infantile GM2-gangliosidosis cerebellar cortex was the exception, increasing in size. Elevated intracranial pressure (estimated by lumbar spinal pressure) was a common finding in infantile disease and showed continued increases as the disease progressed, yet lacked MRI signs of hydrocephalus except for increasing ventricular size. Notably, in patients with juvenile gangliosidosis, macrocephaly and elevated intracranial pressure were absent and total brain volume decreased with time compared to controls (P = 0.004).

**Conclusions:**

The disease course of infantile versus juvenile gangliosidoses is clearly distinguished by the rate of brain disease progression as characterized by qMRI. Assessments by qMRI represent a robust non-invasive method for monitoring CNS changes in the clinical course of gangliosidoses and is ideally suited to monitor effects of novel CNS-directed therapies in future clinical trials.

## 1. Introduction

Infantile and juvenile GM1-gangliosidosis and the GM2-gangliosidoses (Tay-Sachs disease and Sandhoff disease) are inherited metabolic diseases in which deficiency of a lysosomal enzyme results in accumulation of gangliosides in the central nervous system (CNS), leading to progressive neurodegeneration, and early death [1]. In GM1-gangliosidosis, mutations of the GLB1 gene result in the production of defective β-galactosidase enzyme. The GM2-gangliosidoses (Tay-Sachs disease, Sandhoff disease, AB variant and B1 variant) are due to defects that reduce hexosaminidase A enzyme activity. Hexosaminidase A is composed of two subunits, the α-subunit and β-subunit. The formation of a heterodimer between the α-subunit and β-subunit is necessary for activity of hexosaminidase A [1,2]. In Tay-Sachs disease, mutations occurring on the HEXA gene result in a defective α-subunit [1,2]. In Sandhoff disease mutations occurring to the HEXB gene, prevent production of a normal β-subunit [1–3]. In the B1 variant of Tay-Sachs disease, a heterodimer between the α-subunit and β-subunit of hexosaminidase is able to form, but defects in the catalytic site of the hexosaminidase A enzyme result in deficient catalytic activity of the dimer [1,2]. In the AB variant of GM2-gangliosidosis, the genetic defect is caused by mutations of the GM2A gene, which encodes for the ‘activator protein’ of hexosaminidase which acts as a requisite cofactor in ganglioside substrate binding and catalysis. Thus, in the AB variant, normal hexosaminidase enzyme is produced, but is not catalytically active on gangliosides [1–3].

Patients with the infantile forms of GM1- and GM2-gangliosidoses present with similar patterns of neurodevelopmental dysfunction by 6 months of age. Profound hypotonia and inability to sit or hold head up independently are common presenting symptoms. Eye movement abnormalities often occur within the first year of life. Progressive disease involves development of dysphagia, seizures and excessive salivary and respiratory secretions before 18 months of age [1–6]. Macrocephaly, atypical head shapes, and hypomyelination are common in infantile gangliosidoses [7,8]. Death usually occurs between 2.5 and 3.5 years of age, with most patients not living beyond 4 years of age [1–6].

The juvenile forms of GM1- and GM2-gangliosidoses also share similarities in presentation. The onset of symptoms in the juvenile forms of both diseases occurs in early to mid-childhood, usually between the third and fifth year of life. Common presenting symptoms are ataxia and/or dysarthria. Disease progression includes development of dysphagia, progressive hypotonia, and seizures [1,2,4,9,10]. For patients with juvenile GM2-gangliosidosis, death usually occurs in mid-childhood to early adolescence, with most patients not living past the age of 15 years [1–4,6–9]. Lifespan for patients with juvenile GM1-gangliosidosis ranges from mid-childhood to early adulthood [1,2,6,10]. An intermediate form of GM1-gangliosidosis, called late-infantile GM1-gangliosidosis, has been more recently described [6]. Late-infantile GM1-gangliosidosis is considered to be a subset of juvenile GM1-gangliosidosis [6]. In late-infantile GM1-gangliosidosis, symptoms appear usually between one to two years of age, with death in mid to late childhood [6].

There are currently no FDA approved treatments for gangliosidosis diseases. An improved understanding of the natural history of these conditions is necessary in order to advance treatment development. Key to this is a better understanding of how the central nervous system (CNS) changes over time, and the association of CNS changes with progression of motor and cognitive impairments.

The most commonly reported MRI findings in infantile and juvenile gangliosidoses have been dysmyelination, and cerebellar and cerebral atrophy. These finding correspond with the ataxia in the juvenile forms and impaired motor skills and movement disorders in infantile forms. These findings have been documented through clinical case reports and several natural history studies [3–4,6,9–20]. Sequential MRI changes in 6 patients with late-infantile GM1 gangliosidosis and 9 patients with juvenile GM1 gangliosidosis, performed by Regier, et al. showed increased total brain volume, with progressive brain atrophy of the cerebellum and cerebrum, with atrophy being more uniform and more rapidly progressive in the late-infantile form compared to the juvenile form [6].

Although brain atrophy has been well documented in the childhood gangliosidoses, quantitative brain volumetric studies in very young children are lacking. Definitive MRI neuroimaging of brain structures in children < 2 years old is hindered by a number of factors. These include developmental intensity changes in the MRI images that prevent readily distinguishable segmentation of the cortical interface between grey and white matter. Higher levels of cortical ribbon folding create complex shapes that increase difficulty of localization when reading images of very young children. Low tissue contrast leads to difficulties in detecting cortex edge and partial volume effects in select areas of the brain [21]. These age-specific limitations make it difficult to perform fully-automated segmentation techniques, such as using FreeSurfer [22] or FSL [23]. Structural brain abnormalities such as macrocephaly, atypical head shapes, and hypomyelination, common in infantile gangliosidoses [7,8], further complicate the process of brain MR image-registration and normalization, which are essential steps in the automated segmentation pipeline.

## 2. Objectives

In this context, the goal of this longitudinal study has been to quantify and track brain MRI volume changes, including specific structure volume changes, at different times in disease progression of childhood gangliosidoses, and to explore quantitative brain MRI volumetry (qMRI) as a non-invasive marker of disease progression for future treatment trials.

## 3. Methods

Children were enrolled under an IRB-approved process for retrospective chart review and prospective observation. Subject diagnosis was confirmed by leukocyte enzyme activity, and genotyping of GLB1 (GM1-gangliosidosis), HEXA (Tay-Sachs disease) and HEXB (Sandhoff disease). Thirty-five healthy control subjects were scanned twice, one year apart, on a separate IRB approved protocol. Serial brain MRI images, collected approximately one year apart, were evaluated in patients with infantile and juvenile gangliosidoses.

Sagittal T1-weighted MR images were obtained on a 3-Tesla MR scanner (MAGNETOM Skyra and Trio, Siemens Medical Solutions, Erlangen, Germany) with 20-channel head coil array using three-dimensional magnetization-prepared rapid gradient-echo imaging (3D MP RAGE) pulse sequence with TR/TE/TI 2530/3.65/1100 ms, 7 °flip angle, 256× 256 acquisition matrix, 256 mm^2^ FOV, 240 slices, 1.0 mm slice thickness, GRAPPA with acceleration factor of 2, bandwidth 180 Hz/Px, echo spacing 8.5 ms, and an acquisition time of 5 min 53 s. Intracranial volume, brain, and compound ventricular volumes were measured using ITK-SNAP v.2.4.0. ITK-SNAP is a semi-automated 3D image contour segmentation tool controlled by user-defined initialization and region-growing parameters, utilizing snake evolution [24]. The snake is a closed curve representing a segmentation that evolves from a very rough estimate of the anatomical structure of interest to a very close approximation of the structure. To determine intracranial volume we used the edge-based snake, whose evolution slows down near edges or discontinuities of intensity. The region competition snake, which is made to attract to boundaries of regions of uniform intensity, was applied for a segmentation of brain, ventricles, and intracranial volumes. The lateral ventricles and the 3rd and 4th ventricles were segmented separately, and their volumes were added altogether to obtain the compound ventricular volume. Output labels were edited and manually corrected as necessary by an experienced operator (IN). The time required to complete one case was approximately 45–60 min. Changes in cerebellar white matter, cerebellar cortex, caudate, putamen, and corpus callosum were measured by manual tracing using BRAINS2 software [25]. This work was done by an experienced tracer (AA) and the complete analysis of one case took approximately 5 to 6 h. Volumes of left and right caudate nuclei were added together as well as both putamina. Basal ganglia volume was reported as a compound of both caudate nuclei and putamina.

Lumber spinal fluid opening pressure was measured, under general anesthesia at the time of MRI imaging, using a standardized procedure in the lateral recumbent position with end tidal CO_2_ adjusted to be 30–35 mm Hg at the time of assessment. A cerebral spinal fluid (CSF) pressure of > 20 cm was considered evidence of intracranial hypertension [26–27].

Head circumference-for-age percentiles were calculated for occipital-frontal circumference (OFC) measurements using the supplemental z-scores and standard deviations published in the World Health Organization head circumference-for-age growth chart for children aged up to 36 months [28]. For OFC measurements of children > 36 months of age, percentiles were calculated from the median and standard deviations published by Roche, et al. [29]. OFC data was evaluated for presence of macrocephaly or microcephaly, defined as ≥ or ≤ 2 standard deviations above or below the standard reference range, respectively [28–29].

The medical record was reviewed for relevant clinical features such as the child's age at onset of symptoms, the progression of disease symptoms, OFC and morphometrics, medications, history of seizures, operative notes, MRI reports and data files, and all clinical consultation reports.

### 3.1. Statistical methods

Subject characteristics were summarized by group: healthy control, infant GM1, infant GM2, juvenile GM1, and juvenile GM2. A mixed effects linear regression model with random intercept was used to estimate the first-order trend in volumes over time separately for each group and contrast to controls. Five brains were randomly selected and re-analyzed by the same rater (IN) one week after the first analysis to assess reliability of the method, which was assessed with intra-class correlation coefficient (ICC). The relationship between MRI volumes, opening pressure, and OFC was analyzed with Pearson correlation coefficient. All analyses were conducted using R v3.2.4 [30].

## 4. Results

Fourteen patients with childhood gangliosidosis were enrolled: four with infantile GM1-gangliosidosis (1 male, 3 females); two females with juvenile GM1-gangliosidosis; one male with late-infantile GM1-gangliosidosis; five with infantile Tay-Sachs disease (3 females, 2 males); and two females with juvenile Tay-Sachs disease (Tables 1 and 2). No patients with Sandhoff disease participated. No patients with the B1 variant and AB variant of GM2-gangliosidosis participated. Covariates of the 35 healthy control subjects are shown on Table 2.

Eleven of the fourteen subjects had at least two brain MRI exams at approximately one-year intervals and three subjects had one MRI exam. Structural regions of interest were generated in all subjects. Intra-rater ICC was excellent for all measured structures. ICC for intracranial volume (ICV) was 0.98, for brain 0.97, and for compound ventricular volume, 0.96. For manually-traced structures the intra-rater ICC ranged 0.90–0.96.

**Fig. 1:**
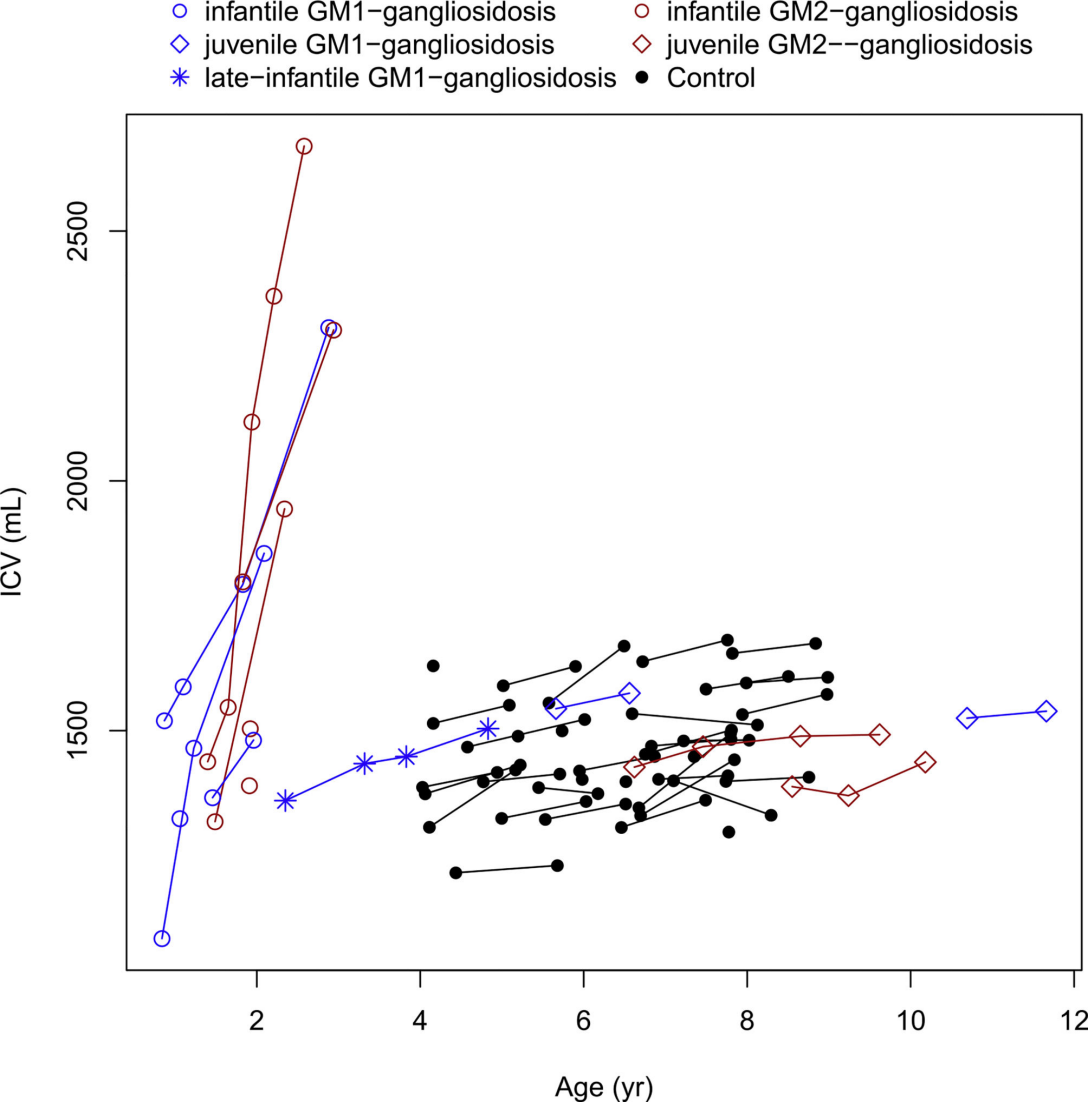
Change in the intracranial volume (ICV) with age.

**Fig. 2:**
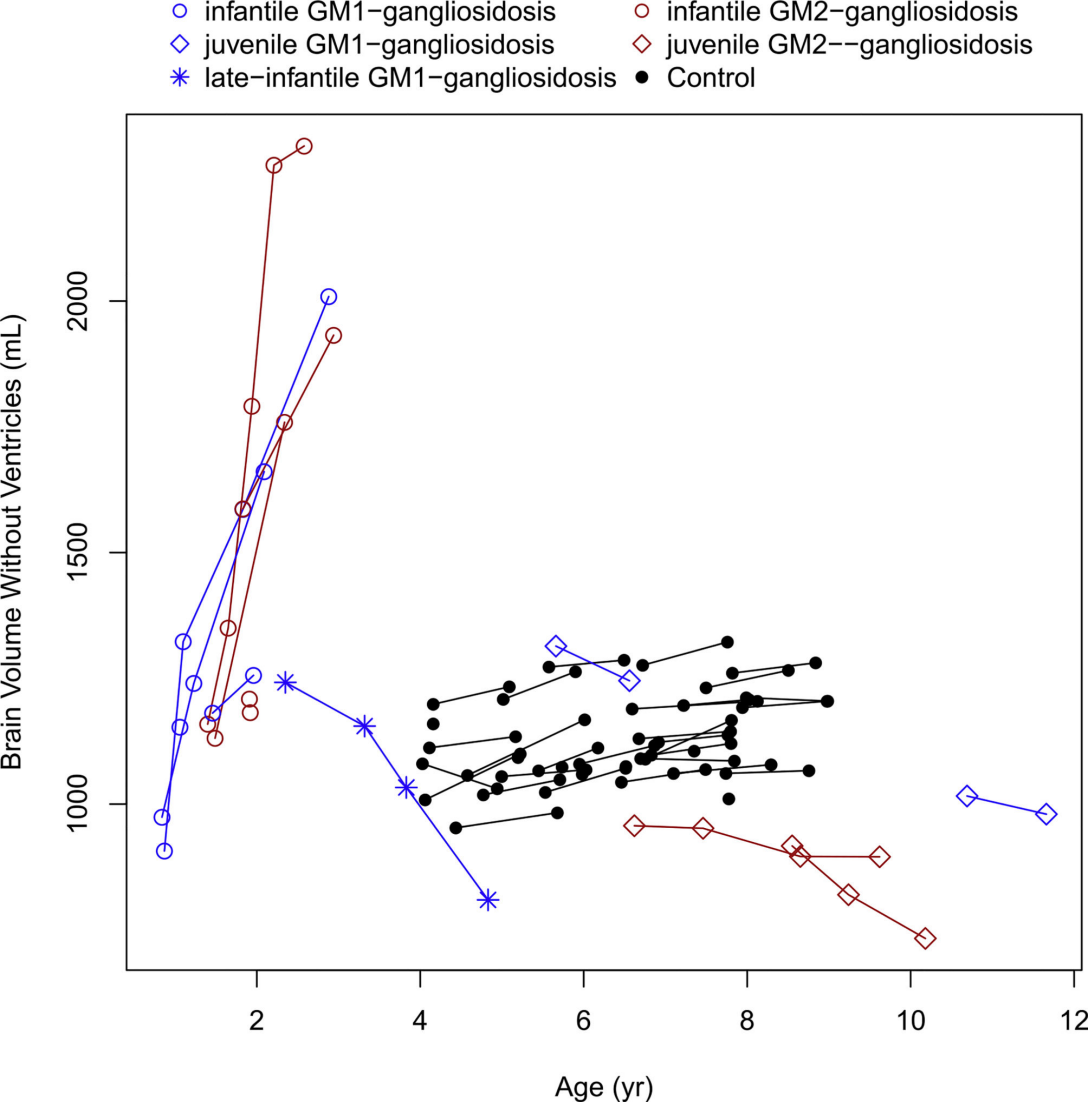
Change in the total brain volume, without ventricular volume, with age.

**Fig. 3:**
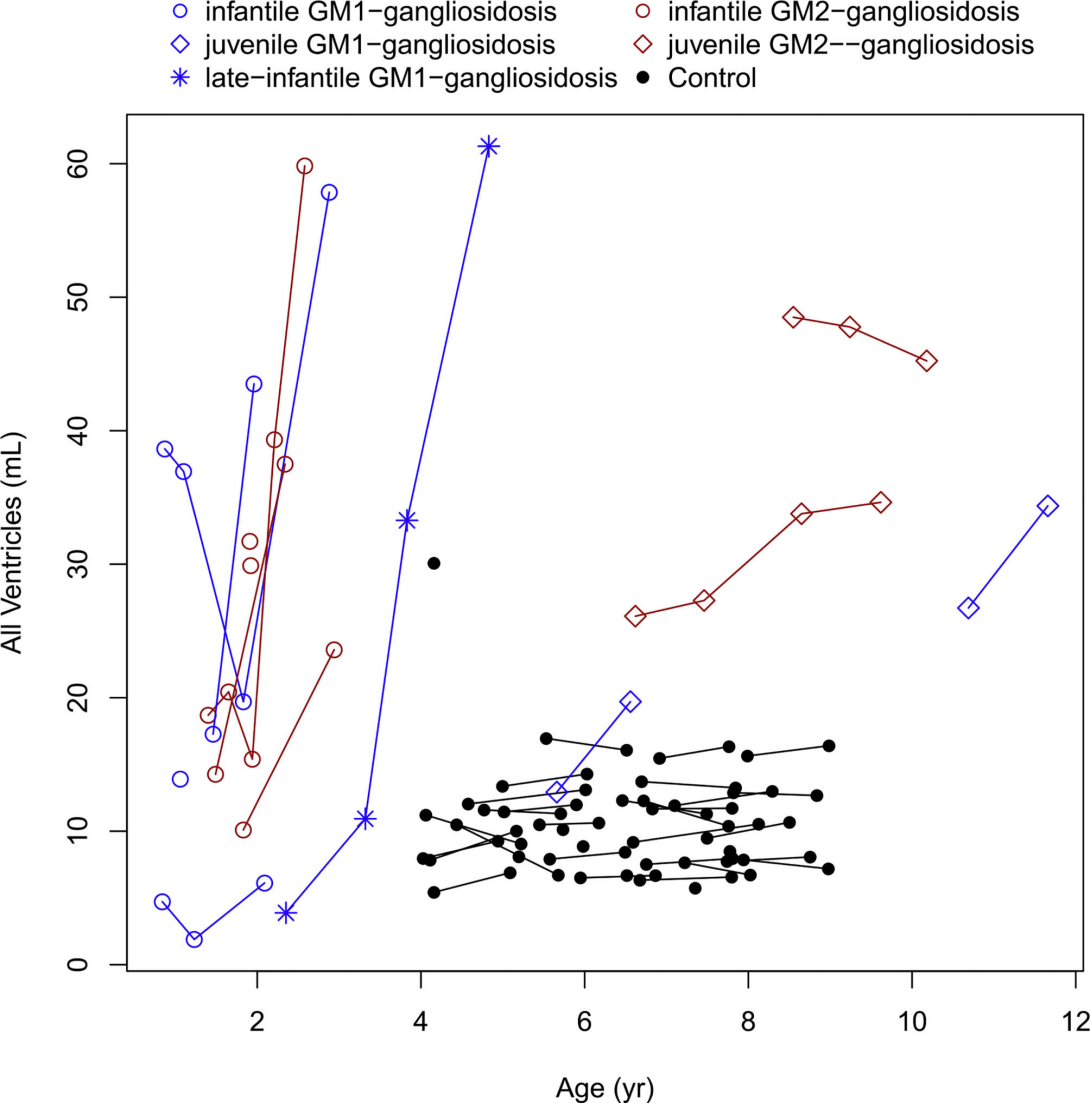
Change in the compound ventricular volume with age.

Intracranial volume, total brain volume without ventricles (cerebral cortex and other smaller structures), and compound ventricular volume showed an abnormal increase in patients with infantile forms of GM1- and GM2- gangliosidoses (Figs. 1, 2, 3; Tables 3 and 4), and these findings were existent before the age of 2 years. The patient with late-infantile GM1 gangliosidosis (subject #10) also showed a rapid increase in ventricular volume, similar in slope to the infantile phenotype, but occurring after 2 years of age (Fig. 1). In contrast, the patients with juvenile gangliosidoses showed relatively stable intracranial volumes that were similar to those of the healthy controls (Fig. 1; Tables 3 and 4). In both juvenile disease groups, there was a mild total brain volume decrease (Fig. 2; Tables 3 and 4) with a stable or mild increase in ventricular volume (Fig. 3; Tables 3 and 4).

**Fig. 4:**
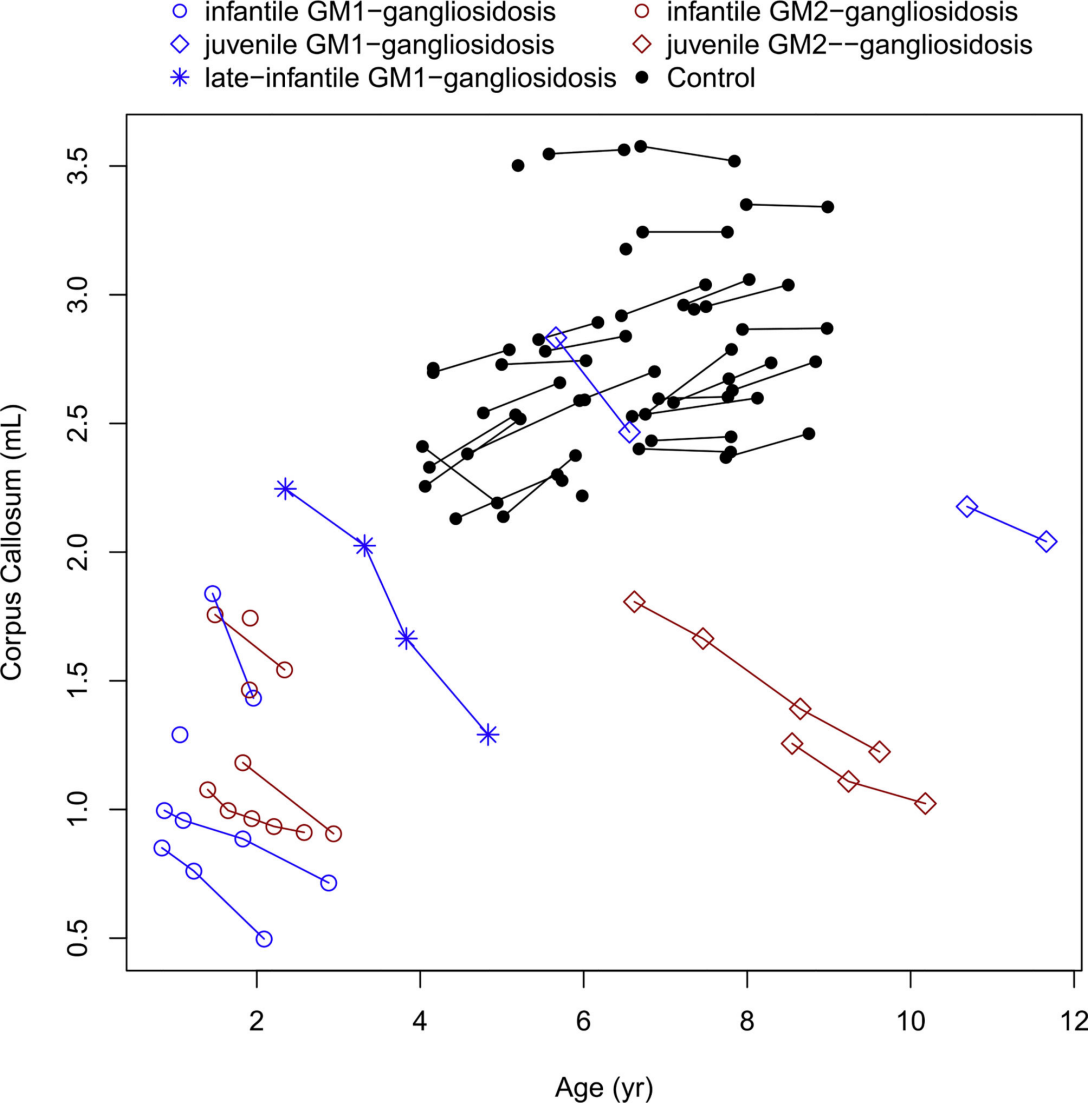
Change in the cerebellar white matter volume with age.

Cerebellar white matter showed gradual decrease in volume over time, in all patients (both infantile and juvenile phenotypes), and was markedly lower compared to the controls (no controls were available for the infantile subjects) (Fig. 4). Moreover, while the cerebellar white matter was gradually decreasing in study subjects, it was increasing in the control group.

**Fig. 5:**
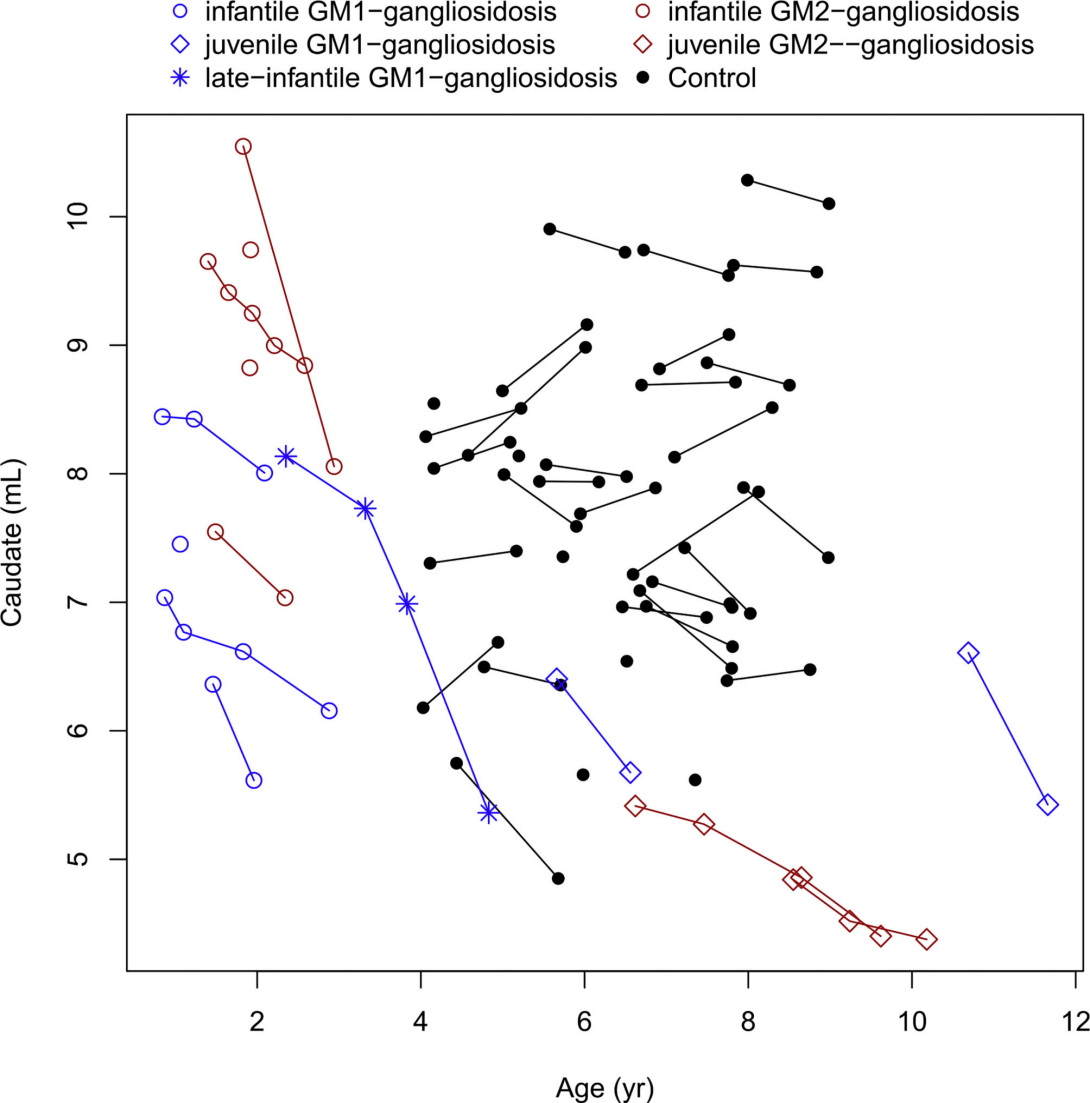
Change in the caudate nuclei volume with age.

**Fig. 6:**
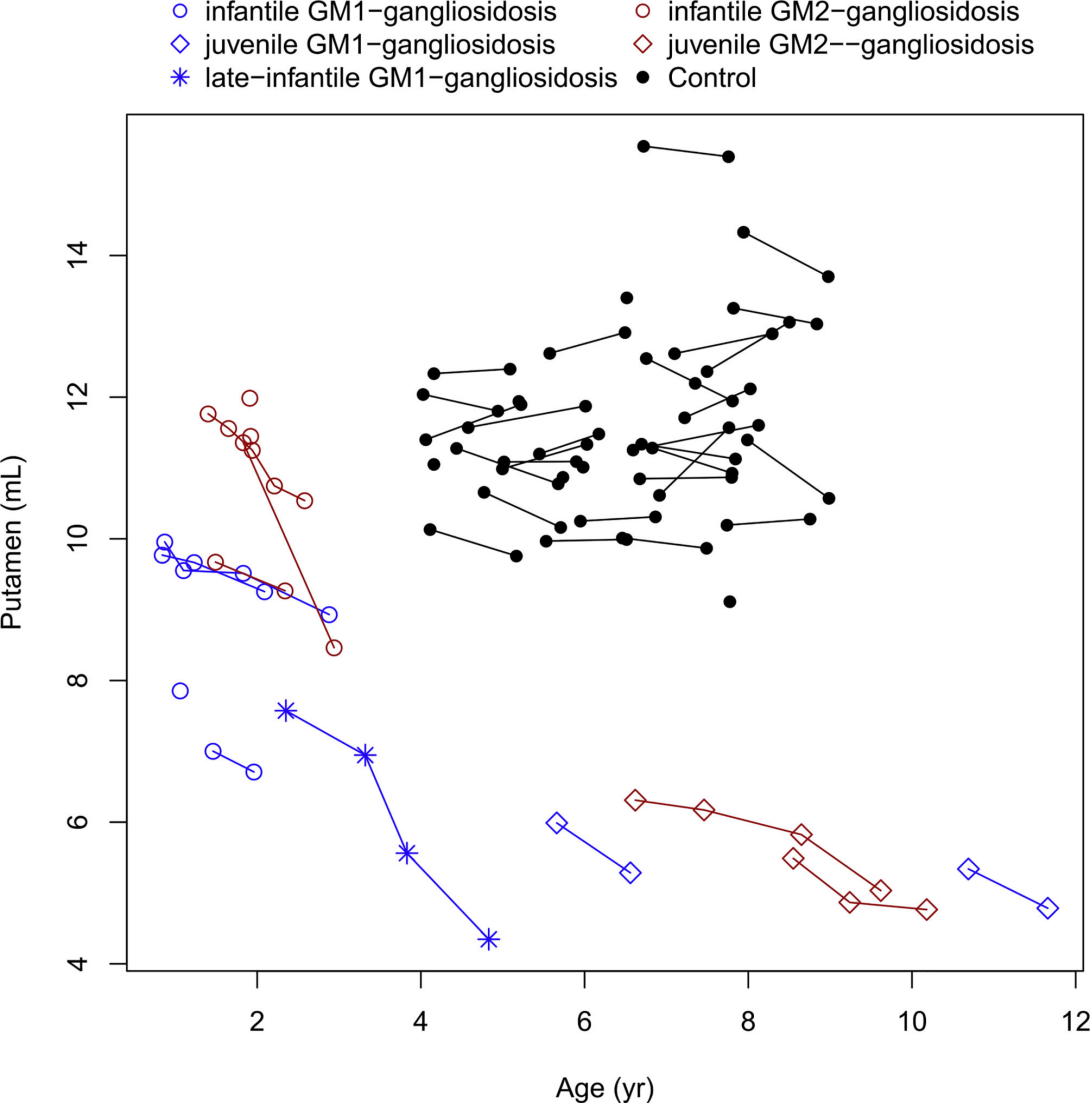
Change in the putaminal volume with age.

**Fig. 7:**
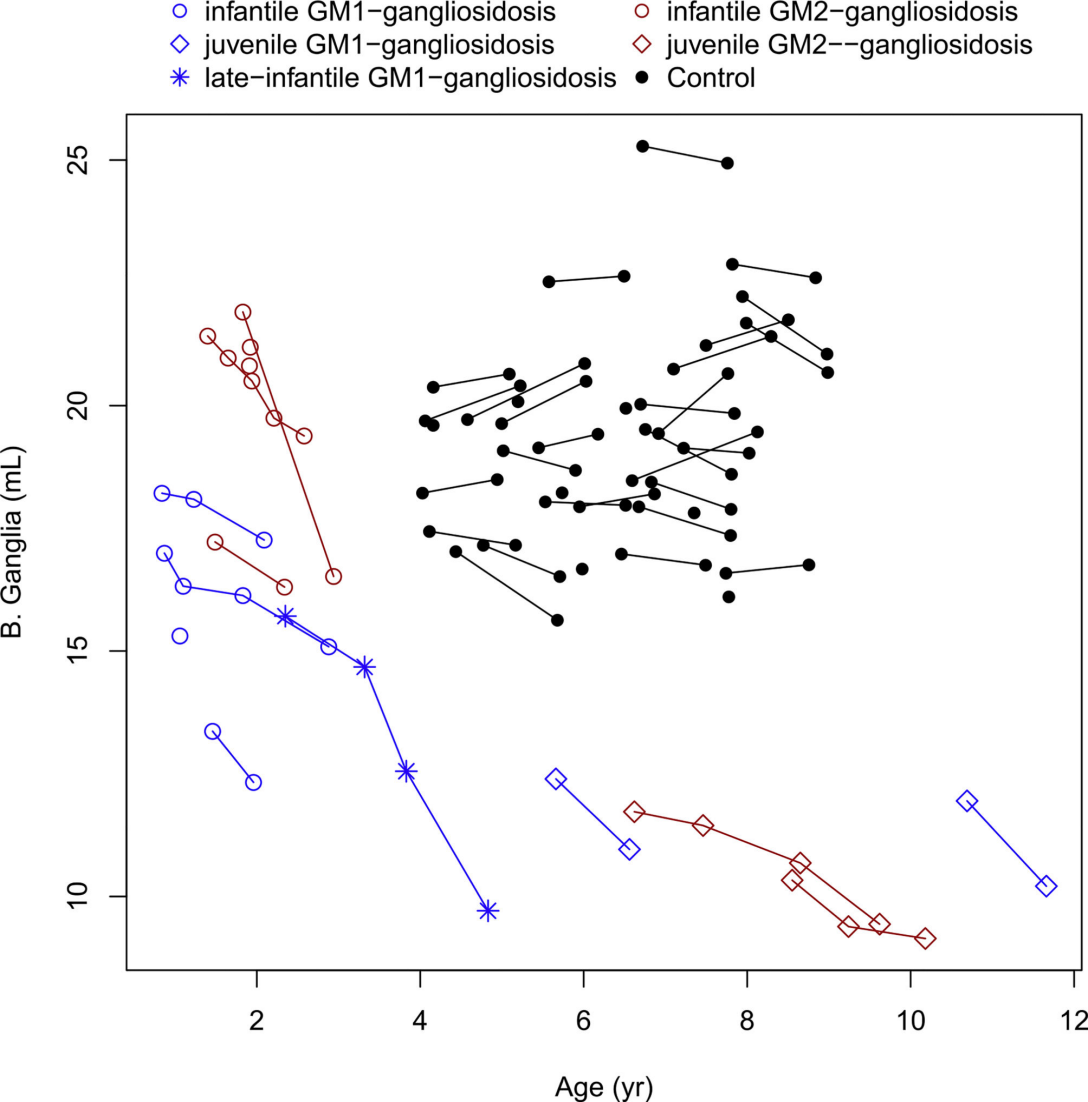
Change in the corpus callosum volume with age.

**Fig. 8:**
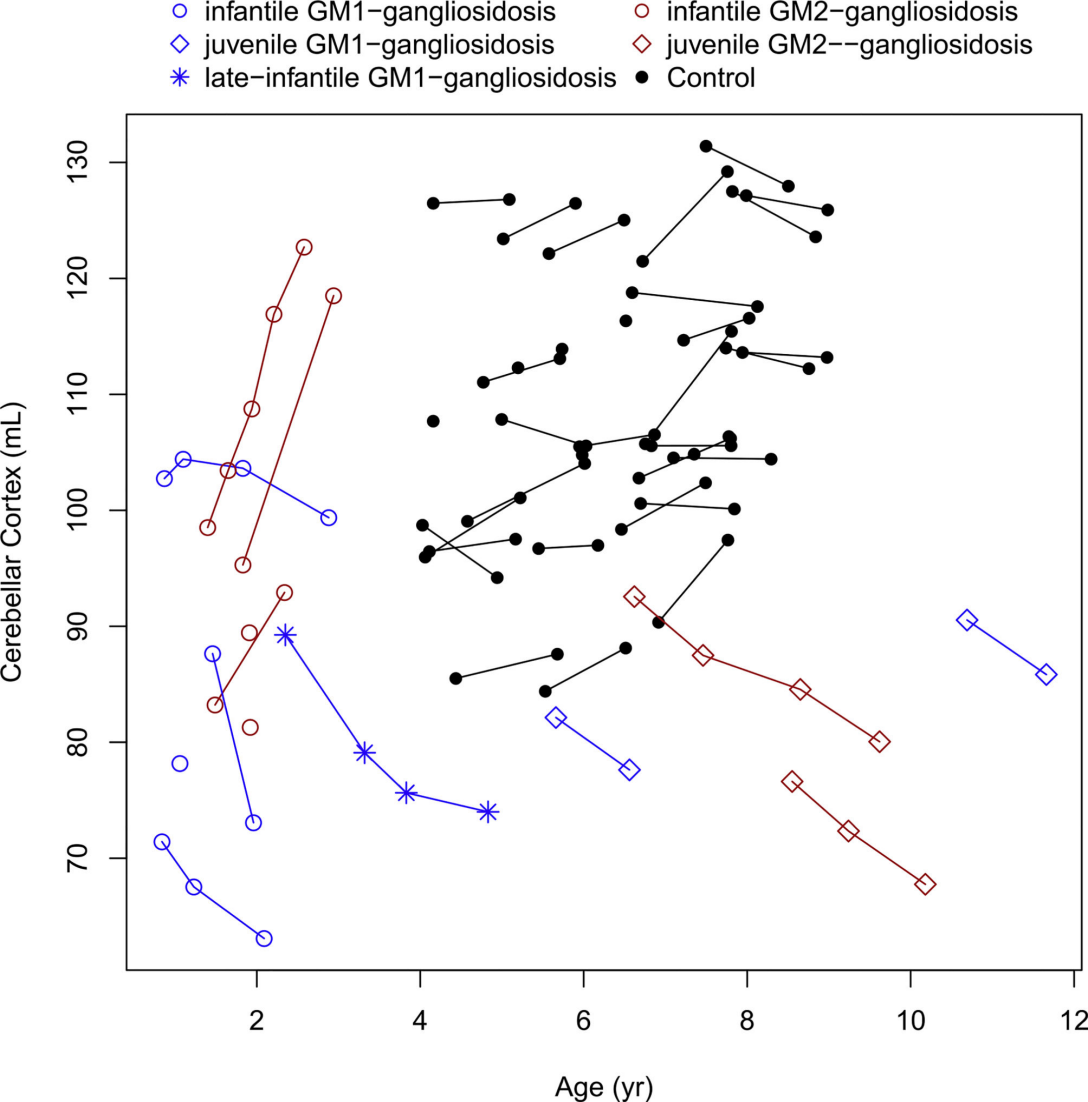
Change in the basal ganglia volume with age.

**Fig. 9:**
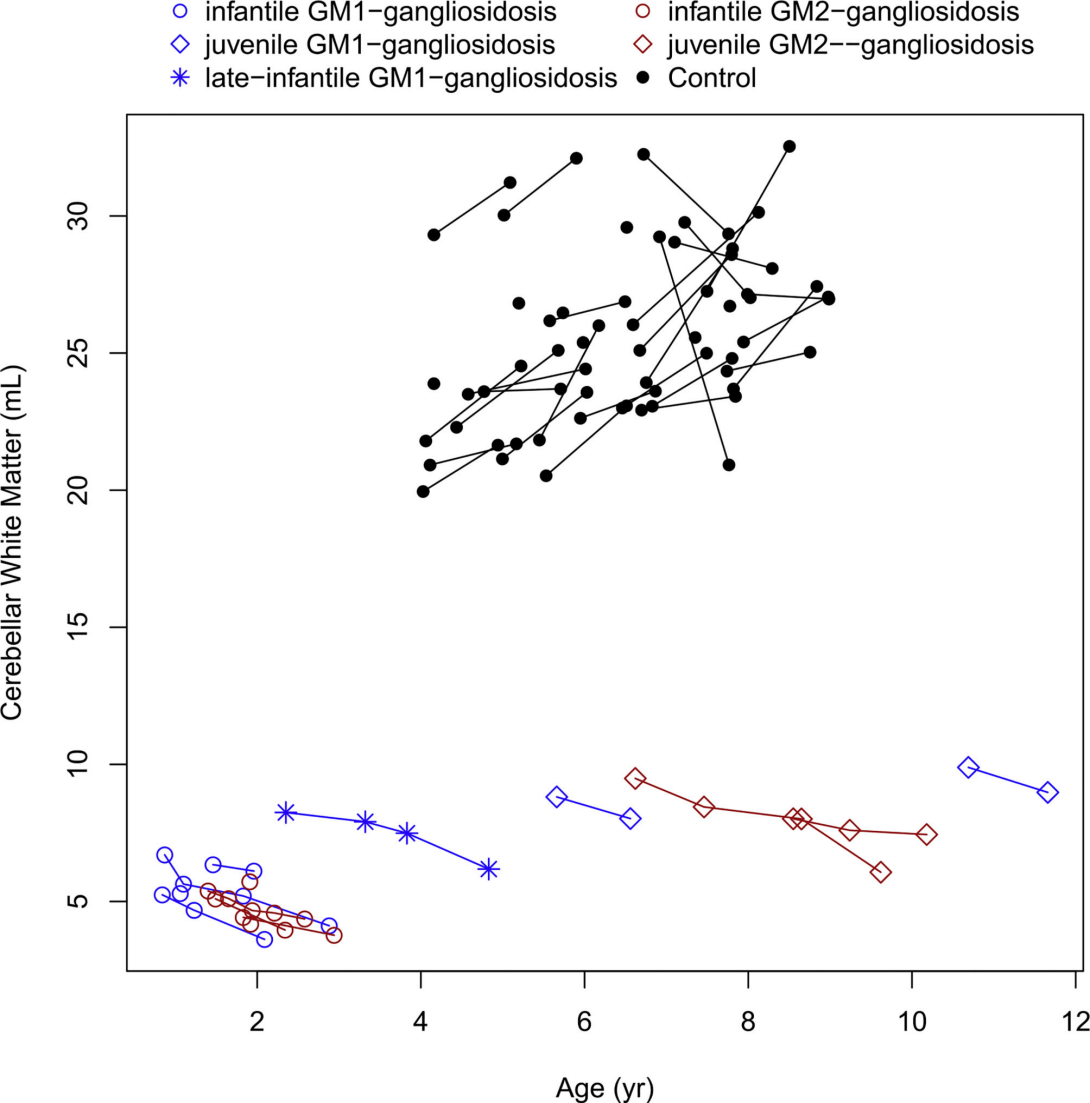
Change in the cerebellar cortex volume with age.

The caudate, putamen, corpus callosum and basal ganglia showed marked decreased over time in all patients (Figs. 5, 6, 7 and 8). These values were markedly low compared to controls, for the patients with juvenile disease. The cerebellar cortex volume decreased in all patients, with the exception of infantile GM2-gangliosidosis (Fig. 9) where we observed a marked increase over time.

One patient with infantile Tay-Sachs disease (subject #5) was observed to have a spontaneous subdural and epidural hemorrhage. Notably, this was not associated with any history of antecedent head trauma, or any measurable abnormality in the coagulation cascade or thrombocytopenia.

In patients with infantile disease forms, head growth as reflected in OFC was markedly abnormal. Beginning as early as 6 months of age, OFCs were measured as > 2 standard deviations above the normal age-specific mean. All nine infantile patients met the classification of macrocephaly (> 2 SD from age specific mean), with 6 patients having OFC in the 99.9th percentile. In contrast, children with juvenile forms of gangliosidoses maintained normal head size throughout the study period.

**Fig. 10:**
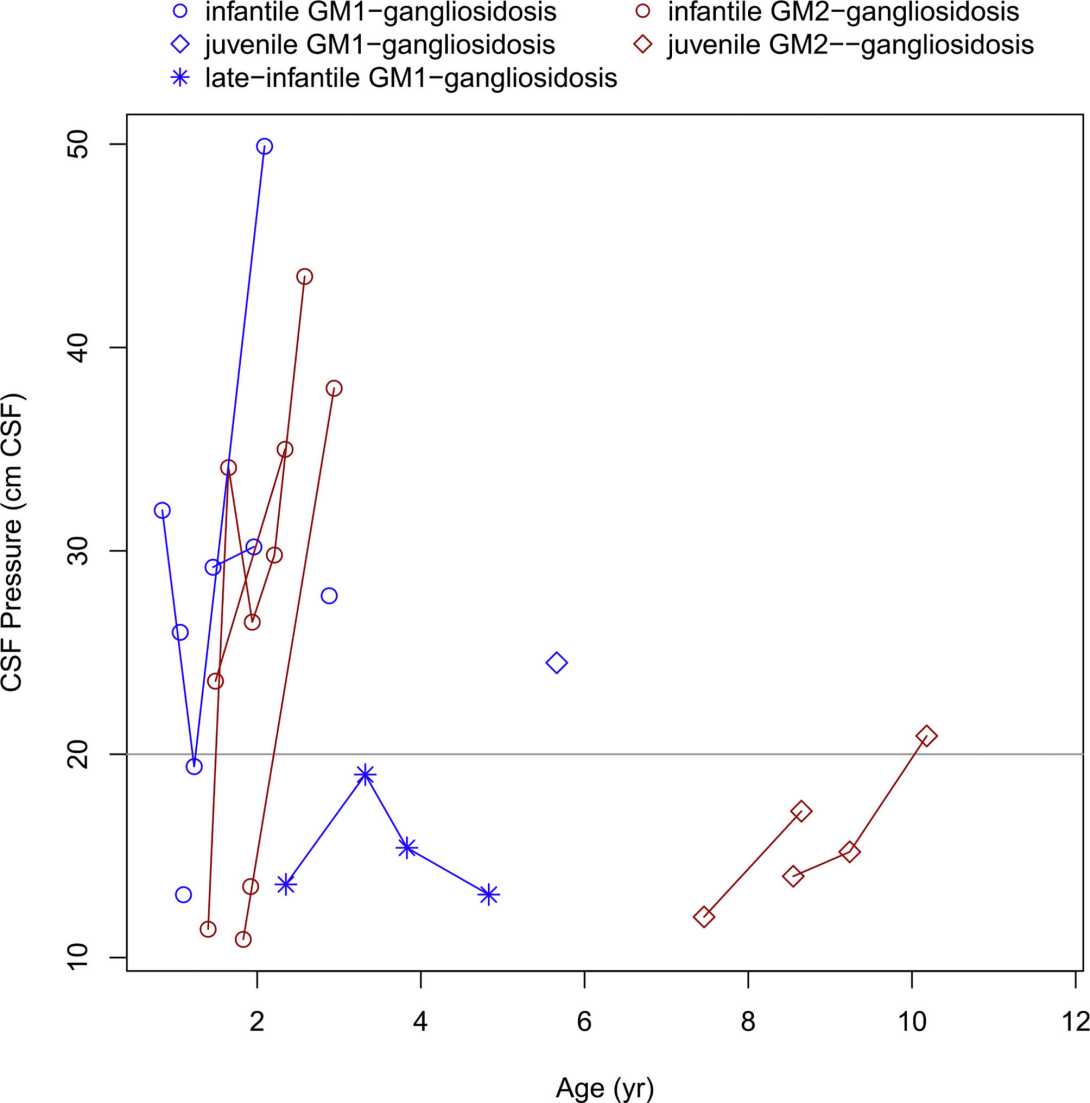
Change in the CSF opening pressure with age.

CSF pressure was elevated in seven of nine patients with an infantile phenotype (Fig. 10), but no patients showed evidence of hydrocephalus on MRI. In patients with juvenile disease (n = 5), none had evidence of increased CSF pressure, with the exception of one subject (juvenile Tay-Sachs) whose pressure was borderline. Correlation between CSF opening pressure and total brain volume (excluding ventricles) was strong (r^2^ = 0.613, 95% CI 0.311, 0.803, P = 0.001). Correlations between CSF opening pressure and ICV (r^2^ = 0.525, 95% CI 0.188, 0.751, P = 0.004) and CSF opening pressure and OFC (r^2^ = 0.473, P = 0.011) were also significant. Correlation between CSF opening pressure and compound ventricular volume was not significant (r^2^ = 0.038, 95% CI −0.405, 0.340, P = 0.847).

In the subject with late-infantile GM1-gangliosidosis (subject #10), considered a subset of the juvenile phenotype, the CSF opening pressure was within normal limits, consistent with the juvenile values. The ICV was comparable to healthy controls, similar to other patients with the juvenile phenotype. The compound ventricular volume was sharply increased, however, and this was in contrast to the other patients with juvenile phenotypes, and resembled the findings of the patients with infantile disease.

## 5. Discussion

This is the first clinical study to use serial quantitative brain MRI volumetry to quantify, and compare between disease and phenotypes within disease groups, the brain structure volumetric changes in infantile and juvenile forms of GM1- and GM2-gangliosidoses. Distinctive differences were found between infantile and juvenile forms of the disease, as well as the late-infantile GM1-gangliosidosis subtype. A progressive increase in total brain volume and intracranial volume, accompanied by a rise of CSF opening pressure were noted in the infantile phenotype (both infantile GM1- and infantile GM2-gangliosidosis) but not in the juvenile phenotypic groups. Juvenile patients showed a more mild-to-moderate progression of brain atrophy, compared to patients with infantile disease, consistent with the ongoing, relatively slower, neurodegeneration and clinical progression in the juvenile phenotype.

Despite phenotypic differences in total brain and intracranial volumes, all patients (both infantile and juvenile GM1- and GM2-gangliosidosis) experienced progressive volume decreases for caudate, putamen, corpus callosum, and cerebellar white matter.

Interestingly, the patients with infantile GM2-gangliosidosis had a sharp increase in cerebellar cortex volume, while all other patients groups showed a decrease in this parameter (Fig. 9). This surprising finding is not yet understood. In a murine model of the GM2 activator deficiency (AB variant of GM2-gangliosidosis), the cerebellar Purkinje cells and glial cells showed abnormal ganglioside storage [31]. The mice presented with impaired balance and motor coordination. It is also noteworthy that the cerebellar granule cell layer of the cerebellar cortex has been found to be a distinct storage site of GM2 ganglioside in the Niemann-Pick disease type C mouse [32]. As there is an increase in cerebral volume in both infantile GM1- and GM2-gangliosidosis due to the ganglioside storage, the cerebellar cortex showed a mismatch between GM1- and GM2-gangliosidosis infantile forms. Based on evidence from the murine model, the GM2-ganglioside storage leads to a specific abnormal increase in cerebellar cortex volume. Nonetheless, the pathogenesis is not clear. These progressive changes in brain structural volumes warrant further study to determine a threshold of volume loss/gain that may be predictive for clinical changes in children with gangliosidoses and that can guide the optimal timing for the best treatment outcomes.

Kasama, et al. on autopsy of patients with infantile, juvenile and late-onset GM1 gangliosidosis, found accumulation of GM1 ganglioside in grey and white matter throughout the brain, but most notably in the caudate and putamen, structures of the basal ganglia [33]. Consistent with these early findings, the basal ganglia have shown marked atrophy in numerous case reports, as well as several natural history studies of both GM1- and GM2-gangliosidoses [6,17,34–36]. This suggests the early prominence of basal ganglia pathology in GM1-gangliosidosis, and the possible association of the relative higher amounts of ganglioside in basal ganglia with initial disease presenting symptoms. Most notable of the early clinical symptoms is hypotonia, which is universally present within the first 6 months of life in the infantile phenotype, as well as abnormal eye movements that are often present during infancy in the infantile phenotype [1,3–6]. Both have been associated with profound basal ganglia pathology [37–38]. In the juvenile gangliosidosis phenotype, loss of words and dysarthria are common early presenting symptoms [4,6,9]. Studies in psychiatric illnesses have found verbal fluency performance to be associated with basal ganglia volume, most specifically the caudate nucleus [39].

The corpus callosum, connecting both cerebral hemispheres, is the largest white matter structure in the normal human brain and is easily recognized on normal brain MRI. The subjects in our study showed marked and progressive reduction in corpus callosum volume in both the infantile and juvenile phenotypes. In brain MRI studies of canine and feline animal models of lysosomal diseases, Hasegawa, et al. (2013) found the corpus callosum difficult to recognize in eight animals with GM-1 gangliosidosis (6 Shiba Inu dogs and 2 domestic shorthair cats) and in four animals with GM-2 gangliosidosis (2 domestic shorthair cats, 2 familial toy poodles) [40]. Thin corpus callosum was also shown in four patients with Sandhoff disease [41] and two patients with infantile GM1-gangliosidosis [42].

The precise factors driving the elevated CSF opening pressure in infantile phenotypes are unknown, but considerations include progressive gliosis occurring in the infantile form in response to progressive brain injury [8]. The rapidly progressive increases in ganglioside storage in the infantile form should also be considered as a possible contributor, especially in light of the dramatic brain volume increases that may be difficult to explain by gliosis alone. We showed that CSF opening pressure increases were not accompanied by MRI signs of hydrocephalus other than *ex vacuo*. Hydrocephalus *ex vacuo* is a condition usually seen as a compensatory enlargement of the CSF spaces in the presence of brain atrophy. The single case of the late-infantile GM1-gangliosidosis patient presented in our study provides additional evidence for this. This patient, with a more attenuated phenotype compared to the infantile form, had normal CSF opening pressure in all assessments, but the compound ventricular volume steeply increased in the presence of diffuse cerebral brain atrophy. Furthermore, there was no association between CSF opening pressure and compound ventricular volumes.

In our study, we used a conventional threshold for elevated CSF opening pressure of 20 cm of water [26–27]. Avery, et al. suggested that the reference range for CSF opening pressure in children should be reconsidered, based on a study of cerebrospinal fluid opening pressure in 472 healthy children between the ages of 1 and 18 years that determined the CSF opening pressure 90th percentile in the healthy study population was 28 cm of water [43]. In our present qMRI study, CSF opening pressure rose above 20 cm and progressed well above 28 cm in all infantile patients for whom sequential measurements were obtained.

One subject with infantile Tay-Sachs disease (subject #6), experienced progressive elevation in CSF pressure and underwent placement of a ventriculo-peritoneal shunt. After shunt placement, the parents reported increased alertness and decreased daytime somnolence. These findings were anecdotal and not conclusive, but reported as part of the clinical natural history.

## 6. Conclusions

This study demonstrates that brain structure volumes assessed by qMRI and changes in these volumes due to disease progression may be quantified in children with gangliosidoses, including children with infantile forms who are < 2 years of age. Further study is needed to determine how thresholds of volume loss/gain, as measured by brain qMRI, correlate with timing and nature of new symptom onset, and how such brain morphometrics may be used to measure clinical outcomes for future therapies. Control values for the infantile phenotype will be required to further define the morphometric changes that are occurring, although obtaining such controls is difficult for numerous ethical and logistical reasons.

In summary, this study demonstrates a new and relatively non-invasive approach to medical surveillance in the infantile and juvenile gangliosidoses. Potential future uses for quantitative MRI volumetry surveillance include provision of critical clinical information on disease progression, measurement of patient response to future central nervous system disease-targeted therapies, and assisting caregivers in anticipating disease complications.

## Figures and Tables

**Table 1:** Individual patient demographics.

Patient (N = 14)	Diagnosis	Gender	No. of visits	Mutation	Age of 1st visit (in year)
1	Infantile GM1	F	4	p.R68W/int 1 5195 ins T	0.87
2	Infantile GM1	F	3	c.1480-2A > G/p.N318D	0.84
3	Infantile GM1	F	1	c.377 T > C/partial del of HEXA gene, extent of deletion undetermined	1.06
4	Infantile GM1	M	2	p.K578R/c.75 + 2dupT	1.46
5	Infantile GM2	F	2	c.929_930delCT/c.1073 + 1G > A	1.83
6	Infantile GM2	M	5	c.1073 + 1G > A/c.1073 + 1G > A	1.4
7	Infantile GM2	F	2	c.986 + 9A > G (intron 8) / c.1073 + 1G > A (intron 9)	1.49
8	Infantile GM2	F	1	c.1073 + 1G > A (intron 9)/partial deletion of HEXA gene	1.92
9	Infantile GM2	M	1	c.508C > T (p.R170W)/c.929_930delCT	1.91
10	Late-infantile GM1 (a subset of the juvenile phenotype)	M	4	p.R201C/p.K578R	2.33
11	Juvenile GM1	F	2	p.L155R/p.L155R	5.66
12	Juvenile GM1	F	2	c.442C > A/c.574 T > C	10.7
13	Juvenile GM2	F	4	c.1274_1277dupTATC/p.G461 V	6.58
14	Juvenile GM2	F	3	c.77G > A/p.R499H	8.55

**Table 2:** Summary of patients' characteristics. Values presented are mean (SD) or N (%) where indicated.

Covariate	Control	Infant GM1	Infant GM2	Juvenile GM1	Juvenile GM2

	(N = 35)	(N = 4)	(N = 5)	(N = 3)^a^	(N = 2)
Male	16 (45.7%)	1 (25.0%)	2 (40.0%)	1 (33.3%)	0 (0.0%)
Age (yr)	6.48 (1.29)	1.46 (0.3)	2.02 (0.21)	6.96 (3.87)	8.71 (0.87)

a Juvenile GM1 group includes late-infantile GM-1 male.

**Table 3:** Rate of change in MRI volumes over time.

Region-of-interest	Group	Change in Volume (mL) per year (95% CI)	P-value
ICV	Cntrl	20.9 (−6.3, 48.2)	0.132
	Inf	564.0 (468.7, 659.4)	**< 0.001**
	Juv	8.1 (−21.0, 37.2)	0.586
Brain (no ventricles)	Cntrl	20.2 (−5.2, 45.6)	0.119
	Inf	558.7 (465.6, 651.9)	**< 0.001**
	Juv	−35.7 (−63.0, −8.5)	**0.010**
All Ventricles	Cntrl	0.00 (−1.68, 1.68)	0.996
	Inf	14.07 (7.75, 20.40)	**< 0.001**
	Juv	2.63 (0.83, 4.42)	**0.004**
Corpus Callosum	Cntrl	0.07 (0.00, 0.14)	**0.042**
	Inf	−0.04 (−0.24, 0.17)	0.732
	Juv	−0.08 (−0.15, −0.01)	**0.025**
Caudate	Cntrl	0.01 (−0.22, 0.23)	0.955
	Inf	−0.17 (−0.88, 0.55)	0.652
	Juv	−0.39 (−0.62, −0.15)	**0.001**
Putamen	Cntrl	0.10 (−0.14, 0.33)	0.423
	Inf	−0.23 (−0.95, 0.49)	0.538
	Juv	−0.16 (−0.39, 0.08)	0.202
Basal Ganglia	Cntrl	0.18 (−0.22, 0.58)	0.374
	Inf	−0.32 (−1.69, 1.05)	0.649
	Juv	−0.54 (−0.97, −0.11)	**0.013**
Cerebellar Cortex	Cntrl	2.06 (−0.16, 4.29)	0.069
	Inf	8.67 (1.88, 15.47)	**0.012**
	Juv	−0.31 (−2.57, 1.95)	0.789
Cerebellar White Matter	Cntrl	0.98 (0.52, 1.43)	**< 0.001**
	Inf	−0.99 (−2.33, 0.35)	0.146
	Juv	0.03 (−0.42, 0.48)	0.895

Cntrl: control. Inf: infantile. Juv: juvenile. ICV: intracranial volume. CI: confidence interval. Significant P-values in bold.

**Table 4:** Difference in rate of change of brain MRI volumes over time compared to control group.

Brain Substructure	Comparison	Difference in Change in Volume per year (95% CI)	P-value
ICV	Inf vs. Cntrl	543.1 (445.1, 641.1)	**< 0.001**
	Juv vs. Cntrl	−12.9 (−53.7, 28.0)	0.537
Brain (no ventricles)	Inf vs. Cntrl	538.5 (442.8, 634.3)	**< 0.001**
	Juv vs. Cntrl	−55.9 (−93.9, −18.0)	**0.004**
All Ventricles	Inf vs. Cntrl	14.07 (7.57, 20.57)	**< 0.001**
	Juv vs. Cntrl	2.62 (0.13, 5.12)	**0.039**
Corpus Callosum	Inf vs. Cntrl	−0.11 (−0.32, 0.11)	0.318
	Juv vs. Cntrl	−0.15 (−0.26, −0.05)	**0.004**
Caudate	Inf vs. Cntrl	−0.17 (−0.91, 0.57)	0.649
	Juv vs. Cntrl	−0.39 (−0.73, −0.06)	**0.022**
Putamen	Inf vs. Cntrl	−0.32 (−1.07, 0.42)	0.395
	Juv vs. Cntrl	−0.25 (−0.60, 0.10)	0.157
Basal Ganglia	Inf vs. Cntrl	−0.50 (−1.91, 0.91)	0.486
	Juv vs. Cntrl	−0.72 (−1.32, −0.12)	**0.019**
Cerebellar Cortex	Inf vs. Cntrl	6.61 (−0.40, 13.62)	0.064
	Juv vs. Cntrl	−2.37 (−5.66, 0.92)	0.158
Cerebellar White Matter	Inf vs. Cntrl	−1.97 (−3.35, −0.59)	**0.005**
	Juv vs. Cntrl	−0.95 (−1.61, −0.28)	**0.005**

Cntrl: control. Inf: infantile. Juv: juvenile. ICV: intracranial volume. CI: confidence interval. Significant P-values in bold.
